# Application of TILLING and EcoTILLING as Reverse Genetic Approaches to Elucidate the Function of Genes in Plants and Animals

**DOI:** 10.2174/138920208784533656

**Published:** 2008-06

**Authors:** N.A Barkley, M.L Wang

**Affiliations:** USDA-ARS, Plant Genetic Resources Conservation Unit (PGRCU), 1109 Experiment Street, Griffin, GA 30223, USA

**Keywords:** Reverse genetics, functional genomics, TILLING (target induced local lesions in genomes), EcoTILLING (Ecotype TILLING), sequencing, SNP (single nucleotide polymorphism), genetic stocks.

## Abstract

With the fairly recent advent of inexpensive, rapid sequencing technologies that continue to improve sequencing efficiency and accuracy, many species of animals, plants, and microbes have annotated genomic information publicly available. The focus on genomics has thus been shifting from the collection of whole sequenced genomes to the study of functional genomics. Reverse genetic approaches have been used for many years to advance from sequence data to the resulting phenotype in an effort to deduce the function of a gene in the species of interest. Many of the currently used approaches (RNAi, gene knockout, site-directed mutagenesis, transposon tagging) rely on the creation of transgenic material, the development of which is not always feasible for many plant or animal species. TILLING is a non-transgenic reverse genetics approach that is applicable to all animal and plant species which can be mutagenized, regardless of its mating / pollinating system, ploidy level, or genome size. This approach requires prior DNA sequence information and takes advantage of a mismatch endonuclease to locate and detect induced mutations. Ultimately, it can provide an allelic series of silent, missense, nonsense, and splice site mutations to examine the effect of various mutations in a gene. TILLING has proven to be a practical, efficient, and an effective approach for functional genomic studies in numerous plant and animal species. EcoTILLING, which is a variant of TILLING, examines natural genetic variation in populations and has been successfully utilized in animals and plants to discover SNPs including rare ones. In this review, TILLING and EcoTILLING techniques, beneficial applications and limitations from plant and animal studies are discussed.

## GENOMICS REVOLUTION

The development of the modern day field of genomics began in 1977 with the publication of two chemical enzymatic DNA sequencing methods developed by Sanger *et al*. [1], and Maxam and Gilbert [2], as well as a publication of the first complete DNA sequence of the bacteriophage, phiX174 [3]. Since 1977, the refinement and automation of the Sanger method of dideoxy chain termination sequencing has led to complete sequencing, assembly, and annotation of an extraordinary number of genes and genomes. The only completely sequenced genomes made available from 1977-1995 were either viral or organelles, which have smaller genome sizes [4] than most plants and animals. In the last decade, due to the progression of sequence automation, computing technology, and bioinformatics, there have been many advances in genomics. This includes a number of model species with their whole genome sequenced. Rapid sequencing has improved over the years and has changed considerably, advancing from a method that has been applied for some time known as the automated Sanger dideoxy chain termination sequencing [4, 5] to now include newer more efficient technologies such as pyrosequencing [6], reversible terminator sequencing [6], ligation sequencing [6], and nanopore sequencing that is currently under development [5]. These technologies continue to improve the efficiency, accuracy, and reduce the cost and time required to sequence large genomes. In the next five to ten years these new rapid sequencing methods will revolutionize the ability of scientists to quickly obtain complete assembled genomic information on many species of animals, plants, and microbes, which will become publicly available to all researchers. 

Currently, the NCBI (National Center for Biotechnology Information) website (www.ncbi.nlm.nih.gov), a resource for molecular information, cites that 668 genomes have been completely assembled from prokaryotes; in contrast, only 339 eukaryotic genome sequencing projects are currently ongoing of which 22 are completely assembled. There are also 1,310 mitochondria and 121 plastids completed and publicly available from eukaryotes. Additionally, 1,959 completely sequenced viral genomes are publicly available, which are the cause of many serious diseases in humans such as HIV and influenza. Since microbes in general have simple and relatively small sized genomes comparatively to eukaryotes, many researchers sequenced these less complicated organisms such as *Agrobacterium tumefaciens *(cause of crown gall disease), *Bacillus spp*., *Campylobacter jejuni *(causes gastroenteritis in humans), *Escherichia coli*, *Fusobacterium nucleatum* (dental plaque), *Helicobacter spp*., *Lactobacillus spp*. (convert lactose to lactic acid), *Mycoplasma spp *(parasites or saprobe), *Propionibacterium acnes* (acne) *Staphylococcus spp*, and *Streptococcus spp*. Some of the animal sequencing projects that are in progress or are currently assembled include simple to very complex organisms such as *Drosophila melanogaster* (fruit fly), *Caenorhabditis elegans* (worm), *Canis lupus familiaris *(dog), *Ciona itestinalis* (sea squirt), *Gallus gallus* (chicken), *Homo sapiens* (human), *Mus musculus* (mouse), *Rattus norvegicus* (rat), *Pan troglodytes* (chimpanzee), and *Takifugu rubripes* (pufferfish). The number of sequenced plant genomes including some agriculturally important crops has lagged behind the sequencing of microbes and animals due to large genome sizes, high proportion of repetitive DNA, and the complication of various ploidy levels such as hexaploid wheat. The first published plant genome available was from the model plant *Arabidopsis thaliana*, but since then others have followed such as *Oryza sativa* (rice), *Populus trichocarpa* (black cottonwood), and *Medicago trunculata*. The plant sequencing projects currently in progress include *Lotus japonicus*, *Solanum lycopersicum *(tomato), *Solanum tuberosum *(potato), *Glycine max* (soybean), *Manihot esculenta* (cassava), *Sorghum bicolor *(sorghum), and *Zea mays *(maize).

Many believed that once many of these genomes were sequenced scientists would be able to better understand genome organization including gene function and ultimately manipulate genes in a genome by genetic engineering. A key target for genetic engineering would be to alleviate disease problems in plants and animals. One of the main problems for determining the mechanism of human disease such as lupus is that epistasis, or the interaction between genes at different loci, can play a major role in disease susceptibility [7]. This interaction between genes can not be detected by knowing the sequence content; therefore, it must be determined empirically. Therefore, pinpointing the mechanism and causes of disease requires more effort than sequencing a specific genome. Another problem is a lack of information on the function of many genes in a particular genome. Bioinformatics and computing technology is helping to unravel the coding DNA and predict gene sites in the genome as well as gene function based on similarity to other deposited sequences, which should provide many insights. However, similarity does not always translate into equivalent function especially when comparing across distant taxa. Furthermore, large portions of sequenced genomes in plants and animals currently have not yet been assigned a putative function based on homology to known proteins [8]. Therefore, other strategies are necessary to empirically identify unknown genes and elucidate their respective function. 

## GENETIC APPROACHES FOR FUNCTIONAL GENOMICS

Two main approaches utilized to link genotype to phenotype are known as forward and reverse genetics. Both of these processes aim to determine the function of a gene / genes through screening the phenotype or genotype of individual mutants to ultimately determine how it is controlled. Traditionally utilized, forward genetics (phenotype to genotype), in which one starts with a particular identified phenotype or biological process and the gene sequence is ultimately deduced through screening large numbers of mutagenized individuals for phenotypic variations is a useful approach. However, forward genetic approaches are not practical for genome wide analysis primarily due to the effort and time involved to identify each gene coding for a particular phenotype [9]. In reverse genetics (from genotype to phenotype), the gene sequence is known and mutants are screened to identify individuals with structural alterations in the gene of interest [10]. This approach is generally less time demanding than forward genetics. Reverse genetic strategies have been successfully used for functional genomics in many animal and plant species. The widespread availability of sequence data allows researchers to rapidly design reverse genetic strategies to determine gene function. Some of the reverse genetic strategies employed in plants and animals include homologous recombination, *Agrobacterium* mediated insertional mutagenesis, transposon tagging, RNAi (RNA interference) or PTGS (post transcriptional gene silencing), and chemical mutagenesis. Even though many of these strategies are effective, they can often be organism specific [11]. An overview and the advantages and disadvantages of these strategies will be discussed.

Homologous recombination is a reverse genetic approach that has become routine for some microbes such as *E. coli* and yeast, but has proven to be much less effective or efficient to employ in multicellular eukaryotes such as plants [8, 12]. Many attempts have been made in various species of plants to optimize homologous recombination with limited success rates [12, 13]. In mice, *Mus musculus*, homologous recombination is a common practice and has been optimized to provide precise mutations in any gene from embryonic stem cells [11, 13]. This procedure is advantageous because it can target specific sites in the genome and disrupt them by reciprocal exchange of DNA. It is thought to occur by double strand breaks, replacement and subsequent repair to ultimately knock out or disrupt the target gene. The disadvantages to this approach include the creation of transgenic material, lack of efficiency, and the limited success rate with many species. 

The gram negative bacterium, *Agrobacterium tumefaciens,* which causes crown gall disease (tumors) in many host plants, has been turned into a widely used tool for reverse genetic research in plant systems. This soil borne bacterium naturally infects wounded plants by transferring T-DNA (located on a plasmid known as Ti for tumor inducing plasmid) from the bacteria to the plant, which subsequently integrates into the plant’s genome [8]. Engineered strains of *Agrobacterium tumefaciens* have been successfully used to create numerous transgenic plants including many agronomical and horticultural important species such as corn, soybeans, and cotton [14]. Many transgenic rice plants have been genetically modified to increase the plant’s ability to resist disease, tolerate salt and cold stress, and have improved nutrition by adding genes encoding for β-carotene biosynthetic pathway to the endosperm allowing the synthesis of vitamin A [15]. Advantages to this approach include generation of large mutation populations that can be created and stored, as is currently available for *Arabidopsis* [12]. Additionally, this approach has the potential for improving nutritional value or disease resistance in crop plants. A disadvantage of this method is inefficient transgene expression [14]. Another potential disadvantage is that in some plants, such as soybean (*Glycine max*), transformation success may be genotype specific [16] and of relatively low efficiency.

Transposons, also known as jumping genes, were first discovered by Barbara McClintock in the 1940’s when she analyzed the mosaic pigmentation patterns of maize kernels. Since their discovery, they have been broadly utilized for reverse genetics studies in plants and animals. In 1995 Pioneer Hi-Bred used the Mutator (*Mu*) transposon on a large scale to screen for *Mu* insertions in genes of interest in maize. One of the potential drawbacks of *Mu* is detection of somatic *Mu* insertions in genes that are thus not transmitted to the offspring [17]. In animals, transposons known as P-elements have been vastly used to examine gene function in *Drosophilia*. These elements have been genetically engineered for insertional mutagensis and stocks that carry an insertion in a gene of interest can be ordered [18]. Advantages include complete or partial disruption of gene function depending on the transposon insertion site. Of course, this reverse genetic approach requires the creation of transgenic material which is often not feasible in certain species.

RNAi has been widely employed and studied in plant and animal systems. RNAi is a gene silencing mechanism, which occurs through the cell’s recognition and degradation of double stranded RNA (dsRNA) and ultimately causes interruption to a gene’s function [19] in diminished protein production. One of the earliest examples of post transcriptional gene silencing (PTGS) in plants was found in petunia where genetic constructs were introduced in an attempt to increase expression levels of chalcone synthase (CHS), which is a key component in flavonoid biosynthesis responsible for plant and flower pigmentation. The experiment unexpectedly resulted in the cosuppression of the endogenous and exogenous CHS gene expression [20]. Research later demonstrated that dsRNA activated the silencing mechanism in *Caenorhabditis elegans* [21]. Most eukaryotes have RNAi capability, and thus, can process dsRNA into small pieces of 22-25 bp. These short fragments can proceed to recognize homologous transcripts for degradation or target genes for DNA methylation [12]. RNAi technology is currently being examined to treat human disease. The main hurdles for RNAi therapeutic treatment for disease in humans is delivery and safety [22]. Advantages in certain organisms include that the silencing is heritable and systemic [23]. Disadvantages of this method include low throughput and efficiency of gene silencing can vary and be unpredictable [12]. 

Since the 1940s, chemical mutagenesis, which was first discovered to produce mutations in *Drosophila* and plants, has long been used as a tool to obtain mutants for reverse genetic approaches [24]. Numerous novel varieties of crop plants such as rice, wheat, cotton, and sunflower have been derived through mutation induction [25]. Chemical agents allow researchers to examine a range of various mutations in a gene of interest including silent, missense, nonsense, splice site, and deletions. These induced mutations can facilitate an association between a genotype and a particular phenotype. Some of the chemical agents used to create mutants include ethylmethane sulfonate (EMS), N-ethyl-N-nitrosourea (ENU), N-nitroso-N-methylurea (NMU), and ionizing radiation. The advantages of chemical mutagens is that they tend to produce a relatively high density of random mutations [12] throughout the genome including gene knockouts. Furthermore, induced point mutations generate a range of alleles for genetic analysis [26] (Tables **1** and **2**). Additionally, the creation of transgenic material is not required. Chemical mutagensis does not require tissue culture and the mutations are heritable in the successive generations [16]. Lastly, unlike other reverse genetic approaches chemical mutagenesis is applicable in most taxa that can be mutagenized. The main disadvantage of chemically induced mutations was an efficient, practical approach for detection. TILLING, a high throughput mutation detection method, takes advantage of chemical mutagensis to generate induced mutations in a population, which results in a high mutational density with very low levels of aneuploidy and dominant lethality [27].

## HISTORY AND OVERVIEW OF THE TILLING METHOD

TILLING first began in the late 1990’s from the effort of a graduate student, Claire McCallum (and collaborators from Fred Hutchinson Cancer Research Center and Howard Hughes Medical Institute), who worked on characterizing the function of two chromomethylase genes in Arabidopsis [28]. Claire McCallum utilized reverse genetic approaches such as T-DNA lines and antisense RNA, but was unable to successfully apply these approaches to characterize CMT2. The approach that was successful turned out to be what is now known as TILLING (Targeting Induced Local Lesions in Genomes). This was accomplished by pooling chemically induced mutagenized plants together, amplifying the region of interest, creating heteroduplexes among the pooled DNA, and performing dHPLC (denaturing high performance liquid chromatography) to detect the mutants by chromatographic alterations [29]. Since the inception of this method, TILLING has been streamlined, automated, and utilized in many plant and animal taxa.

**Fig. (1):**
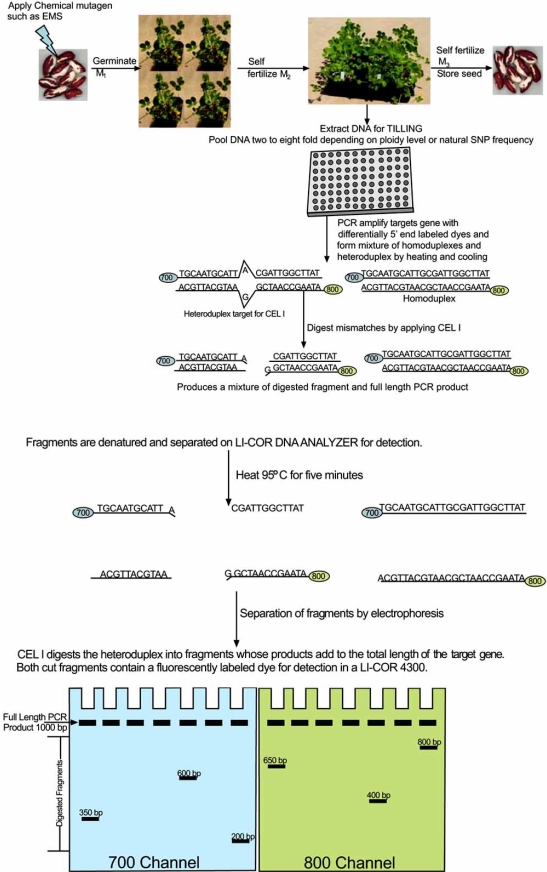
Diagram of the TILLING method in which seeds are mutagenized with a chemical mutagen and germinated to produce M_1_ plants. M_1_ plants are selfed to produce the M_2_ from which DNA is extracted for analysis. The M_2_ is allowed to produce seed which can be easily stored for future analysis. Once the DNA is extracted from the mutant population, the DNA is normalized and pooled together. The number of individuals in a pool depends on the ploidy level of the plant and the amount of naturally occurring SNPs, which may require the number of individuals in the pool to be reduced. The targeted gene is amplified using a forward primer with 700 nm dye label and a reverse primer with an 800 nm dye label attached to the 5’ ends. The PCR products are heated and cooled to form heteroduplexes between the accessions in the pool. The resulting pool will contain a mixture of homoduplexes and heteroduplexes. Any mismatches (SNPs or small INDELS) will be detected by a mismatch endonuclease (CEL I) and cleaved into two separate products, which will be detected in the 700 and 800 dye channel of a LI-COR DNA Analyzer. The additive size of the cleaved fragments should equal the total length of the entire product. Once the cleaved fragments and their respective polymorphic site are identified, these individuals are sequenced to verify the induced mutation. EcoTILLING is performed in the same manner except that the seed are not mutagenized; therefore, the process begins by extracting DNA from a reference plant and members of the population and continuing with the remaining steps to determine natural polymorphisms.

TILLING, which is a reverse genetic high throughput approach, aims to identify SNPs (single nucleotide polymorphisms) and / or INDELS (insertions / deletions) in a gene / genes of interest from a mutagenized population. Therefore, the first step in TILLING is the creation of a mutagenized population, which is often accomplished by treatment with a chemical mutagen such as EMS (Fig. **1**). Many plant species are well suited for this strategy because they can be self-fertilized and seeds can be stored for long periods of time [28, 30]; however, multiple strategies also exist for the creation of mutant populations in animal species [31-33]. In plants, seeds are treated with EMS and grown out to produce M1 plants, which are subsequently self-fertilized to produce the M2 generation. Leaf tissues from M2 plants are collected for DNA extraction and then used for mutational screening [34]. To avoid sampling of the same mutation only one M2 individual from each M1 is chosen for DNA extraction [35]. The M2 progeny can be self-fertilized and the resulting M3 seed can be preserved in long term storage [36]. EMS has been widely used as a chemical mutagen in TILLING in both plant and animal studies to generate mutant populations, although other chemical mutagens can be effective (Table **1**). EMS typically produces transition mutations (G/C : A/T) because it alkylates G residues [37] and the alkylated G residue pairs with T instead of the conventional base pairing with C [12]. It is a beneficial strategy for users to try a range of concentrations of the chemical mutagen being applied to evaluate the toxicity and sterility on germinal tissue before preparing large mutant populations [36].

Once the population has been prepared, the genomic DNA targets need to be selected. The web based program CODDLE (http://www.proweb.org/input) allows users to input genomic, cDNA, or protein sequences and evaluates the probable effect of induced or natural polymorphisms on gene function [26]. Optimal PCR primers can also be designed for a functional domain target. The next step is to collect DNA from the population and normalize the DNA concentration (Fig. **1**). It is crucial to ensure that all DNA samples are equivalent so that no biasing of samples occurs. Once samples have been normalized, they can be pooled together. In general for diploid organisms, a pool of DNA can contain up to eight individual samples in the pool and be successful in mutation detection. With a larger pool size, the sensitivity of mutation detection will decrease, because the proportion of heteroduplexes compared to homoduplexes in the reaction is reduced [12]. Therefore, depending on ploidy level, heterozygosity, and the amount of naturally occurring SNPs, optimal pooling for a species of interest should be determined empirically.

Once the pooled DNA is arrayed into 96 well microtiter plates, pooled samples are amplified using primers targeting the gene of interest. The forward and reverse primers are differentially 5’ end labeled with IRD700 and IRD800 dye labels for fluorescent detection at ~700 nm and ~800 nm, respectively (Fig. **1**). Next, heteroduplexes and homoduplexes are formed from the PCR products of pooled samples (consisting of mutants and the wild type) by heating (denaturing) and cooling (annealing). The endonuclease enzyme CEL I is applied and a short incubation is required for the enzymatic reaction to progress. CEL I, isolated from celery, not only specifically recognizes mismatches in the heteroduplex, but it also cleaves DNA on the 3’ side of the mismatch [30]. After the enzyme incubation period, detection of any digested fragments occurs by separation on a denaturing polyacrylamide gel attached to a LI-COR 4300 DNA analysis system (Fig. **1**). Pools containing an induced mutation will consist of a mixture of homo- and heteroduplexes. Therefore, when fragments are separated a full length product (detected in both 700 and 800 channels) and two cleaved fragments (one IRD700 labeled, one IRD800 labeled) will be visible. The sum of the cleaved fragments should equal the full length PCR product. The size of the cleaved fragments can be estimated by comparison to a size standard, and thus, the approximate location of the mutation will be identified and further confirmed by sequencing. The web based program PARSESNP http://www.proweb.org/parsesnp/ can be used once mutations are identified to display the locations of the polymorphisms in a gene/ genes in a graphical format [38]. (A protocol and materials required for TILLING in Arabidopsis using a LI-COR DNA analyzer has been published [39] as well as protocols for preparing celery juice extract for enzymatic digestion [40] and two pooling strategies [41]). 

## TILLING IN PLANTS 

Since the inception of TILLING, this method has been widely used for the study of functional genomics in plants, especially for the model plant *Arabidopsis thaliana*. In 2003, Greene *et al*. reported that the *Arabidopsis* TILLING Project (ATP), which was set up and introduced as a public service for the *Arabidopsis* community [36], had detected 1,890 mutations in 192 target gene fragments. Heterozygote mutations were detected at twice the rate of homozygote mutations [27]. Therefore, the mutational density for treatment of *Arabidopsis* with EMS was approximately 1 mutation / 300 kb of DNA screened with these mutations distributed throughout the genome [27]. The numerous mutations in *Arabidopsis* *thaliana* that have been identified *via *TILLING have provided an allelic series of phenotypes and genotypes to elucidate gene and protein function throughout the genome for *Arabidopsis* researchers.

Another model plant, *Lotus japonicus*, has also been the focus of elucidating gene function through TILLING. *Lotus japonicus* is a perennial temperate legume that is a model plant for genomic studies because it has a short life cycle, is a diploid (2*n* = 2*x* = 12), has a relatively small genome (472 Mb), and is self fertilized [42]. TILLING was used to investigate induced mutations occurring in the protein kinase domain of the SYMRK gene, which is necessary for root symbiosis [43]. Six missense mutations were identified along with a mutation in the splice acceptor site [43]. Nitrogen fixation and the functional role of sucrose synthase was the target of another *Lotus japonicus* TILLING study [44]. Six isoforms of sucrose synthase were identified and several mutations including missense and nonsense were located in four of the six isoforms. Quantitative RT-PCR was performed to examine expression levels in *L. japonicus*, which were determined to have differential expression in various plant organs. Furthermore, EMS null allele mutants were examined and shown to have reduced sucrose synthase activity compared to the wild type; however, mutants still retained the ability for nitrogen fixation [44]. In a separate study of pea (*Pisum sativum*), which also fixes nitrogen and is a member of the legume family, TILLING was applied to identify an allelic series of mutations in five genes with a total of 60 mutants identified [45]. Some of the mutations discovered in the LE gene, which encodes 3β-hydroxylase, were further characterized and determined to affect internode length. Mutants were backcrossed to the wild type and the segregation of the mutations and their respective phenotypes were examined [45].

Due to the success of ATP (now known as Seattle TILLING Project, STP), TILLING has radiated from the model plants such as *Arabidopsis* with a simple small diploid genome (125 Mb) to other agronomically important crop plants with more complex genomes. In 2004, maize, which is an important staple crop with a large genome, was shown to be conducive to the TILLING method [46]. A total of 11 genes were examined in a population of 750 mutagenized plants and six of these 11 genes had detectable induced mutations. One of the genes examined in this study, DMT102 (chromomethylase gene), has been previously suggested to play a role in non-CpG DNA methylation and gene silencing in *Arabidopsis *[46]. A Maize TILLING Project established in 2005 at Purdue University has already identified 319 mutations in 62 genes, which has greatly assisted functional genomic studies in maize [47]. Barley, which is also an important cereal crop with a fairly large genome size of ~5,300 Mb, was evaluated for the ability of induced mutations to be detected by TILLING [48]. Two genes (*Hin-a* and *HvFor1*) were examined and 10 variants were identified, six of which were missense mutations. Phenotyping the M_3_ individuals demonstrated that 20% had visible phenotypes [48].

Wheat is an extremely important agronomic staple crop with an estimated production level of 600 million tons per year [49]. A polyploid plant investigation to locate variants in the *waxy* locus (granule-bound starch synthase I, GBSSI) in wheat was implemented [50, 51]. Partial *waxy* wheat cultivars are desirable because production of amylose starch is reduced, which leads to the production of superior flour and noodle products [52] for human consumption. Wheat genetics can be complicated because its genome is complex, it is an allohexaploid, and the total genome size is quite large (17,000 Mb). A total of 246 alleles were uncovered in three *waxy* gene homoeologues (*Wx*-A1, *Wx*-B1, and *Wx*-D1) from allohexaploid and allotetraploid wheat *via *TILLING. This comprehensive allelic series provided 84 missense, three nonsense and five splice site mutations (Table **2**). Phenotyping of M_3_ progeny demonstrated reduction of amylose production. Detecting genetic variants *via* phenotyping in wheat can be difficult because redundant copies of loci in the genome can mask expression. This study identified more extensive allelic variation in GBSSI than was identified in any report produced in the last 25 years [50, 51].

Rice, which is also a staple and important economic crop around the world, currently estimated to provide 80% of the caloric intake for three billion people [53], has been the focus of a few TILLING studies. The rice genome has been predicted to contain ~50,000 genes [54], of which gene function needs to be determined empirically. In 2005, a report was published on the generation of a large mutation population (60,000) using multiple chemical mutagens on IR64, a widely grown *indica* rice [54]. This study demonstrated that TILLING was suitable for reverse genetic studies with mutations detected in two genes; albeit, the mutational density in the population was fairly low. In addition, extensive phenotypic variation was assessed for the various chemical mutagens used to develop the mutant population and albinism was a common phenotype no matter which mutagen was applied [54]. In a separate study, EMS and Az-MNU were used to induce an elevated mutational density in rice (Table **1**), with 57 polymorphisms identified from 10 target genes by TILLING [35]. Another report on rice TILLING published in 2007, demonstrated the efficacy of TILLING to detect mutations by separation of products on agarose gels [55]. Results were analogous to pooling DNA and detecting mutations on a LI-COR DNA Analyzer [55].

Soybean (*Glycine max*) contains approximately 35-50% protein and has been shown to be beneficial for human health [56]. It is a important economic crop that can improve soil quality by fixing nitrogen [16]. Four mutant populations from two genetic backgrounds (Forrest and Williams 82) were created for soybean by treatment with EMS or NMU and evaluated for induced mutations [16]. Several of the target genes initially tested amplified more than one target. Further work was carried out to produce a single product to employ TILLING so that mutation detection functioned optimally. A total of 116 mutations were identified *via *TILLING from seven target genes. The majority of the mutations uncovered by TILLING were determined to be the expected G/C to A/T transitions. This study demonstrated that soybean is suitable for TILLING studies [16].

Even though TILLING was originally designed for and applied mainly in *Arabidopsis* in the early years of its inception, it has been demonstrated to be an extremely versatile approach compared to many other reverse genetic approaches. This method has proven to be successful to rapidly identify variant genotypes and determine gene function in plants that are diploid and have relatively small genomes such as *Arabidopsis*. Additionally, it also can be easily applied to other crop plants with very large genomes that are further complicated by various ploidy levels such as wheat. Moreover, the use of chemical mutagens in diploid and polyploid plants produce a range of various mutations (Table **2**) and a high density of mutations throughout the genome (Table **1**). Obtaining an allelic series for a gene of interest greatly assists in the overall determination of gene function by providing multiple types of phenotypic variants to be analyzed.

## TILLING IN ANIMALS

Several model animals such as *Drosophila melanogaster*, zebrafish, rats, and *C. elegans*, which have been historically used to investigate numerous genetic hypotheses, have been subjugated to mutagensis for TILLING experiments to gain insight on functional genomics. Unlike plant TILLING, in which mutant populations (in self pollinated species) can generally be easily created by chemical mutagensis followed by self fertilization and harvesting of seed (Fig. **1**), animals require different tactics to establish and maintain mutant populations. In *C. elegans*, if self-fertilizing hermaphrodites are selected, then the creation of a mutant population is fairly similar to plants. The nematode is mutagenized, animals are allowed to self replicate and DNA is extracted for analysis. However, since the majority of animal species can not self fertilize, outcrossing subsequent to mutagensis may be obligatory. No single strategy is applicable or practical to employ for all animal species. Therefore, multiple strategies exist for the creation, maintenance, preservation and recovery of animal mutant populations. An appropriate approach should be chosen based on the life history or availability of tissue preservation / recovery protocols available for the animal of interest.

Since the early 1900’s with the work of geneticist Thomas Hunt Morgan, *Drosophila* has been a well studied model organism for genetic research. Currently, complete genomic information is available for 12 separate species classified in the genus *Drosophila *[57]. This information allows a critical examination of comparative genomics between species [57] and facilitates reverse genetic studies. A commonly used mechanism to evaluate gene function in *Drosophila* is insertional mutagenesis *via *genetic engineering of transposable elements [58]. Homologous recombination is also available to researchers, but is considered a less favorable method [59]. EMS has also been widely used to evaluate numerous genes in *Drosophila* [60]. EMS mutagenesis was used to screen for mutations in the *awd* (abnormal wing discs) gene from *Drosophila* with 16 mutations all G:C to A:T transitions identified by dHPLC [61]. A range of silent, missense, and intron based mutations were detected in this study (Table **2**), as well as a noted mutagensis bias for 5’-PuG-3’ sites or a middle G base in a stretch of three or more G bases [61]. CEL I based TILLING was applied to pinpoint 44 mutations in three genes in a separate study [60]. One of the targets in this study was determined to be an essential gene with several of the induced mutations causing lethality. Winkler *et al*. (2005) found that increasing the EMS treatment by 1.5 fold augmented the frequency of SNPs detected by 1.5 fold; however, this treatment greatly decreased fly viability [60]. A general TILLING service for the *Drosophila* community has been established [37], which helps streamline the effort required for a researcher to determine function in their gene of interest. 

Zebrafish is an aquatic vertebrate model organism, particularly valuable for studying embryonic development. They are easy to maintain and breed, produce a large number of offspring, and females can produce ~15,000-35,000 eggs in their lifetime [62]. Several reverse genetic approaches are available for functional genomic studies in zebrafish. However, sequencing the entire genome is still in production [62], which can be a limiting factor for functional genomics. TILLING has been effective in zebrafish and two separate methods of creating and maintaining mutant populations are published [31, 32]. The first TILLING study in zebrafish reported the creation of 4,608 mutants with ENU that were maintained as a live library [32]. TILLING uncovered 255 mutations in 16 genes of which ~20% of the mutations were found in noncoding sequences. The mutations detected in the coding regions consisted of 119 missense and 14 nonsense [32], all of which should affect the protein function or produce a loss of function. In a separate study, 1,235 mutagenized zebrafish, which were archived by cryopreservation of dissected male testes, were examined *via *TILLING and resequencing for polymorphism in 54 exons (derived from 17 genes) with targets ranging in size from 74 to 626 bp [31]. Polymorphic sites in the genes examined ranged from zero to 26 per amplicon. Due to a high frequency of SNPs in some amplicons, detection of rare induced mutations became somewhat of a challenge [31].

*Caenorhabditis elegans *(soil nematode) is a model organism that has its complete genome sequenced, which has been publicly available for approximately ten years. This genomic information has allowed reverse genetic techniques to be applied to gain insight on functional genomics. Available reverse genetic methods include RNAi, trimethylpsoralen and UV radiation (TMP/UV), transposon insertional mutagenesis and homologous recombination, all of which have certain advantages and disadvantages [33]. *C. elegans* is predicted to contain approximately 19,000 genes [63]; however, mutant alleles are only available for 3,400 genes [33]. TILLING can be used as an effective way to gain an allelic series of mutants in the remaining genes to determine gene function. In 2006, a study examined 1,500 EMS mutagenized nematodes in 10 genes with a total of 71 mutations identified, many of which were determined to have an effect on the protein product [33]. Once mutants were identified *via *TILLING, phenotyping analyses were performed to further characterize or substantiate gene function [33].

Another study examined the feasibility of TILLING in rat [64]. Male rats were treated with ENU, which caused a range of sterile to completely fertile animals to be produced. These chemically treated males were crossed with untreated females to produce progeny that were analyzed by TILLING. A total of 768 animals were analyzed and 17 induced mutations were detected of which 70.5% were missense. Rats were further bred to homozygosity and determined to have germ-line mutations due to Mendelian inheritance of these mutations. Hip dysplasia and diphallus were observed in the mutant population [64]. 

Lastly, the pathogenic bacterium *Photobacterium damselae* subsp. *piscicida*, which is known to cause pseudo-tuberculosis in yellowtail and amberjack fish, has generally been controlled in fish farms with treatment of a quinolone, such as nalidixic acid [65]. Mutant bacteria that demonstrated varying levels of quinolone resistance were pooled with a quinolone susceptible strain for TILLING analysis. The quinolone resistant determining region (QRDR) of the *gyr*A gene was targeted. A single point mutation in all resistant mutants was identified. Although this study was performed on pathogenic bacteria, the results will provide a rapid detection method for resistant bacteria, the avoidance of pointless quinolone treatments to control outbreak of the disease, and possibly prevent economic loss at fish farms [65]. This study not only examined gene function, but also provided data that could be used as a functional marker to rapidly screen pathogenic bacteria and prevent economic loss.

## EcoTILLING IN PLANTS AND ANIMALS

EcoTILLING is a molecular technique that is similar to TILLING, except that its objective is to uncover natural genetic variation as opposed to induced mutations (Fig. **1**). Many species are not amenable to chemical mutagenesis; therefore, EcoTILLING can aid in the discovery of natural variants and their putative gene function [26]. This approach allows one to rapidly screen through many samples with a gene of interest to identify naturally occurring SNPs and / or small INDELS. The method has proven to be successful to detect DNA polymorphisms including variations in satellite repeat number [41]. Furthermore, in highly heterozygous outcrossing species, EcoTILLING can be used to determine heterozygosity levels within a gene fragment [26]. EcoTILLIING reduces the time and effort for SNP discovery generally required by weeding out identical haplotypes. Therefore, this method does not require one to sequence all individuals in a population to identify polymorphisms, which can be a burdensome expense and time consuming. It also has the advantage of detecting multiple polymorphisms in a single fragment because CEL I will digest only a small proportion of the heteroduplexes at a single position [41]. This technique has not been as widely employed as TILLING; however, there are a few published studies on EcoTILLING, which will be further discussed.

The first publication of the EcoTILLING method in which TILLING was modified to mine for natural polymorphisms was in 2004 from work in *Arabidopsis thaliana *[66]. The strategy used was to pool each ecotype with the standard Columbia ecotype (reference) in a 1:1 ratio. A total of 192 accessions were assayed to uncover 55 haplotypes in five different genes that were approximately 1 Kb in length. A large proportion of the variation was detected in the introns. This study demonstrated that CEL I could detect SNPs, INDELS and polymorphisms in microsatellite repeats. Interestingly, a 21 bp deletion was also shown to be detected and cleaved by CEL I enzyme. Overall, EcoTILLING proved to be an efficient method to haplotype individuals without requiring sequencing of all the individuals included in this study [66]. 

EcoTILLING has also been used to examine DNA variation in natural populations of black cottonwood (*Populus trichocarpa*), which is one of the first large deciduous trees to have its genome completely sequenced [67]. DNA polymorphisms were assessed from 41 trees derived from 41 populations originating from Canada to Oregon. Nine separate mapped loci were evaluated by mixing the members of the population to a reference genotype. Each gene produced some polymorphic sites with the number of SNPs ranging from 1-23 and an overall mean of 6.78. The rate of SNPs for coding and noncoding regions assayed were 1/229 bp and 1/64 bp, respectively. Additionally, this study was the first to successfully report the evaluation of heterozygosity of single individual trees with EcoTILLING. Moreover, nucleotide diversity estimates, selection, and linkage disequilibrium were evaluated [67]. This study demonstrated how effective EcoTILLING can be for the evaluation of diversity in natural populations.

Another valuable application of EcoTILLING is mining for variation in resistance genes to help speed up the process of identifying alleles that could provide immunity to various diseases. In 2006, allelic variation was examined and identified by using EcoTILLING in *mlo* and *Mla* resistance genes of *Hordeum vulgare* (barley) [68]. These genes are involved in defending the plant from the fungal pathogen that causes powdery mildew. This study demonstrated that, compared to classical methods of determining disease resistance alleles, EcoTILLING provided several allelic variants in two resistance genes that can be exploited to breed cultivars with improved resistance [68]. In a similar study, EcoTILLING was employed to screen for natural allelic variation for disease resistance to Melon Necrotic Spot Virus (MNSV) in various *Cucumis species* [69]. High conservation of eIF4E, a translation initiation factor, was found among 113 accessions evaluated. Six polymorphisms were identified; however, only one site produced an amino acid change that correlated with disease resistance [69]. In general, EcoTILLING shows great promise of accelerating the process of identifying natural disease resistance alleles, which can be used to breed improved cultivars. 

**Fig. (2):**
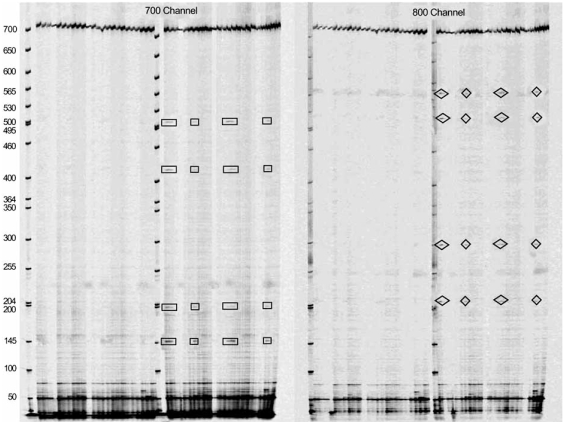
EcoTILLING images produced from a collection of *Vigna radiata*. Polymorphic sites are marked with boxes and were subsequently confirmed by sequencing. A size standard (50-700 bp) was included to estimate the size of the cleaved fragments and the target gene.

EcoTILLING was employed to identify polymorphisms in a germplasm collection of mung bean (*Vigna radiata* (L.) R. Wilczek), which previously showed limited diversity (Barkley *et al. *submitted data). *Vigna radiata*, which is classified in the family Fabaceae, is an important economic crop and a dietary staple in many developing countries [70]. The species *radiata* can be further subdivided into botanical varieties such as *radiata* and *sublobata*, of which *sublobata* is currently acknowledged as the putative progenitor of *radiata* [71, 72]. Polymorphic sites were abundant when comparing *V. radiata *var. *sublobata* to *V. radiata *var. *radiata*; however, when accessions of *V. radiata* var. *radiata *were pooled together relatively few polymorphisms were identified. This suggests that accessions classified as *V. radiata *var. *radiata *could have a narrow genetic base (Fig. **2**). Morphological data taken from accessions of *V. radiata *var. *radiata* also demonstrated limited diversity in the flowers and pod descriptors. The majority of polymorphisms detected between *sublobata* and *radiata* were found in putative introns. The banding patterns varied from simple to complex as the number of DNA polymorphisms between two pooled samples increased. Overall, this modification of the TILLING method has proven to identify natural genetic variation in a gene of interest and to mine for SNPs in plants. Furthermore, it can be effectively used as an efficient, rapid technique to identify DNA polymorphisms in populations with high genetic identity and to mine for SNPs in collections of plant germplasm (Barkley *et al. *submitted data).

In a pioneering animal study, genotypic variation from humans was analyzed by EcoTILLING to detect rare SNPs [73]. Current resequencing efforts for the detection of SNPs in humans tend to identify common polymorphisms not the rare variation that exists in the genome. Detecting rare SNPs can be difficult and quite expensive using standard sequencing methods. In total, 384 human samples were evaluated in five target genes using the EcoTILLING method. This effort produced the detection of 28 rare SNPs, some of which were predicted to have a damaging effect to the protein. This study also examined the precision of EcoTILLING for the detection of SNPs by comparing the SNP dataset detected through public resequencing efforts to those identified by EcoTILLING. Of the 25 SNPs previously identified through resequencing, EcoTILLING uncovered 24 of the 25 [73]. Seven new alleles were discovered, which demonstrates the precision and efficiency of this technology for the detection of rare polymorphisms. 

## ADVANTAGES AND DISADVANTAGES OF TILLING AND EcoTILLING

TILLING is a non-transgenic, high throughput reverse genetic approach. This technique unlike other SNP detection methods, provides the approximate location within a few base pairs of the induced mutation [28, 34], which allows targeted sequencing in the area of the induced mutation opposed to sequencing the entire fragment. Since chemical mutagensis produces a range of various mutations throughout the genome such as nonsense, splice site, and missense, all of which potentially can affect the protein structure and the resulting phenotype, it has been used for decades to obtain mutants for genetic studies. Therefore, through mutagensis one can obtain partial loss or complete loss of function and new novel functions, which can provide valuable insight into the true role of a gene [11] in a species of interest. As discussed previously, using chemical mutagensis and TILLING to pinpoint these mutations has been highly effective in the elucidation of gene function in plants and animals without the production of transgenic material. TILLING has been demonstrated to be sensitive enough to detect induced mutations and naturally occurring SNPs [62], as well as the detection of heterozygotes. EcoTILLING, which has been less frequently employed in the current literature, can also be a valuable tool for mining for SNPs in germplasm, assessing heterozygosity, uncovering variants for disease resistance, or ascertaining the function of a gene or regulatory element by detecting natural variants. EcoTILLING can be a good technique to employ especially when working with a well established population with thoroughly characterized morphological data.

One of the main advantages of TILLING is the amount of time and money this method can potentially save by not requiring resequencing of all individuals in a population to mine for frequent or rare SNPs. As a general rule for a diploid organism, TILLING is performed by pooling eight individuals of a population at a time and assessing differences by endonuclease digestion of mismatches in a heteroduplex. Ordinarily, the majority of the samples screened in TILLING have the same haplotype with very few samples in the population having an induced mutation in the gene of interest due to a relatively low frequency of induced mutants by utilizing chemical mutagenesis (Table **1**). Furthermore, TILLING is sensitive enough to detect homozygous mutations as well as heterozygous mutations in an 8 fold pool, which represent 1 of the 16 genomes in pools from diploid species [34, 46, 50]. This method allows one to weed out the identical individuals and only focus resources on sequencing individuals with rare chemically induced DNA polymorphisms. EcoTILLING also shares this same advantage as a technique except that it focuses on naturally occurring variation as opposed to induced variation as in TILLING. Most EcoTILLING experiments have used a two fold pool strategy [66, 67, 69]; however, one study in which rare human nucleotide differences were examined an eight fold pool strategy was employed [73].

In our EcoTILLING study of mung bean, a two fold pooling strategy was applied as opposed to a four, six or eight fold pooling strategy as often used in TILLING experiments. This was done because multiple SNPs and / or INDELS occurred between individuals in the population. Unfortunately, there is not an efficient strategy available to determine which of the samples in the pool multiple cleaved fragments are derived without remixing the pooled samples and testing various combinations of two fold pools. For example, if one has eight accessions with multiple SNPs, then identifying accessions with SNPs would require evaluating 28 two fold pools. Alternatively, one could mix two, four fold pools (pool 1: samples 1-4; pool 2: samples 5-8) Eco-TILL and possible eliminate one of the four fold pools. Next, remix the positive four fold pool again into six different two fold pools, to cover every possible combination, and Eco-TILL to identify the positive samples. One of the potential disadvantages of EcoTILLING is that when the number of polymorphic sites is high for a gene or PCR fragment of interest across all samples in the population, then an eight fold pooling strategy requires much more labor and time to identify SNPs. This is because eight fold pooling (with a high SNP frequency) would require remixing of numerous pools to locate positive individuals. The other strategy would be to only analyze two fold pools, which requires more PCR reactions, polyacrylamide gels, and increased cost for the mismatch enzyme, but less labor and time remixing samples into new pools to identify positives as would be required for an eight fold pooling strategy. This becomes less of a disadvantage and more malleable to eight fold pooling as the number of polymorphic sites decreases such as EcoTILLING in highly conserved genes or dealing with rare SNPs. Therefore, a potential disadvantage of EcoTILLING is that when variation is high, efficiency of the technique is decreased.

False negatives and false positives can be a potential disadvantage to any reverse genetic application including TILLING and EcoTILLING. This issue has not been extensively examined in the current literature. A study of EcoTILLING of human SNPs did report a fairly low false negative rate of 5% and false positive rate of 4% [73]. False negatives and false positives often can be due to human error in scoring gel images. Generally, this becomes less of a problem as researchers become more experienced in scoring these images. One strategy to avoid this problem is to sequence a small percentage of pools determined to be negative to verify if any false negatives are occurring. An advantage for TILLING and EcoTILLING is that the method helps guard against false positives by double-end labeling the target so that a cleaved product should produce a fragment in both the 700 nm and 800 nm fluorescent channel [27]. Also, the sizes of the cleaved products should total the full length product. In addition, two dimensional arraying/pooling previously described [41, 73], in which samples are mixed in duplicate both by column and by row, also helps reduce false positives. Cleaved fragments produced from an induced mutant are replicated, and thus, will appear in two separate lanes in a gel. This pooling strategy when used in TILLING experiments or EcoTILLING with very low SNP frequencies will also allow one to identify the positive sample carrying the mutation without having to remix the positive pool [41].

One of the initial large expenses of TILLING and EcoTILLING experiments are the use of robotic equipment and the purchase of automated sequencers such as the LI-COR DNA Analyzer commonly used for cleaved fragment detection. However, these products are not essential and experiments can be carried out without the use of robotics. Capillary electrophoresis or other automated sequencers such as ABI 377 have been demonstrated in previous studies to be effective in the separation and detection of digested fragments [43, 68, 74, 75] in lieu of a LI-COR DNA Analyzer. Moreover, PCR products and cleaved fragments can be separated by more economical methods such as agarose gels as reported previously for rice [55] or possibly detection *via *silver staining polyacrylamide gels for fragment separation, which has not been reported yet. The only disadvantage of using agarose gels would be lower resolution between fragments that are similar in size in comparison to the resolving power of polyacrylamide gels. An advantage to using agarose or silver stained gels is that fluorescently labeled primers would not be required, which would remove an additional expense of the technique. If automated sequencers are already purchased or available, one can reduce the experimental costs by purchasing labeled primers using a universal primer strategy [32, 60, 73], in which all reactions are fluorescently labeled with the same primer such as M13 or T7. Lastly, one could also reduce the expense in these experiments by purifying CEL I for endonuclease mismatch detection from celery [40, 41, 68]. Therefore, these techniques can be adapted to less expensive common laboratory equipment, and thus, possible for all researchers to employ.

## TECHNICAL CHALLENGES IN THE APPLICATION OF TILLING AND EcoTILLING 

There are some technical challenges in employing TILLING or EcoTILLING experiments; however, most of these challenges are not insurmountable. The creation of a mutant population can be somewhat of a challenge and sufficient time needs to be allocated for the development of a high-quality population. One to two years can be expected to be devoted to population development [48, 50]. The first step to creating a population is to vary the concentration of the chemical mutagen being applied to assess lethality and to find an optimal concentration to generate a high density of mutants with few lethal embryos [12, 36]. This should be done before too much time is invested in the creation of the entire population. This can be a little bit of a challenge because species and varieties of a species can respond differently to chemical mutagenesis so that a dose of mutagen applied in barley may be highly lethal, but the same dose in *Arabidopsis* will not produce a highly lethal effect [35]. An ideal population would maximize mutational load, but still allow the majority of the population to remain fertile [47] and viable. Creating mutant populations for plants that propagate vegetatively or have long generation times [50] could also slow down progress of generating a mutant population. Working with species that are highly heterozygous may limit mutation detection because lots of cleaved fragments are produced due to natural polymorphisms, which may inhibit detection of rare induced mutations [41]. Variation can be minimized by choosing a single parent to produce thousands of progeny in a single or multiple generations [37].

In most plants, production and maintenance of seeds to preserve mutants for future analysis is fairly straightforward; however, the collection and maintenance of gametes in animals can be somewhat problematic [11]. Therefore, multiple strategies exist for the creation and maintenance of animal mutant populations [11, 31-33]. One must carefully examine the species of interest and decide on a suitable strategy. In animal studies, maintaining live animals as a strategy to preserve the library of mutants as a living resource for analysis [32] can require a large amount of space and labor for the caretaking of the animals. A living mutant resource may be the only option if prior effective cryopreservation methods have not been established for the species of interest such as reported in *Drosophila* and rat [11, 64]. In zebrafish, caudal fins regenerate so that DNA can be extracted from live fish, which can be maintained for a limited time for breeding when mutations are identified [11]. A limitation to a living library is that the library must be screened within the generation time of the animal [32], which may not be an issue in animals that have fairly long life spans, but can be a limitation in animals with short life spans especially if a large number of genes are targeted in a study. 

Once a suitable mutant population, whether in plants or animals, has been established, high quality DNA needs to be extracted and normalized. It is important that all DNA extracts be equivalent so that they are all equally represented in the pools being analyzed. Otherwise, unique induced mutations may fail to be identified, because as the amount of individual DNA decreases in comparison to others in a pool, the sensitivity of mutation detection decreases [46]. Another challenge, especially in regards to plants, can be choosing target genes that only exist as a single copy within the genome. This becomes more of a problem when working with polyploid plants that have multiple genomes such as wheat or peanut, and thus may contain homoeologous genes. Overcoming this challenge can be accomplished by designing primers that are specific to a single gene, which may require some additional effort [76, 77]. Another strategy is to sequence the multiple homoeologous target genes and determine any restriction site differences between the targets. The DNA can be digested prior to TILLING, which would cleave the unwanted target leaving the desired gene intact [16] for analysis. TILLING in polyploid plants generally requires a reduction of the number of samples mixed in a pool [46, 51], so throughput may be somewhat decreased in comparison to diploids. This is because recognition of mismatches becomes more difficult with an increasing number of genomes. Detection of a heterozygous mutation in a tetraploid or hexaploid individual in an eight fold pool could be overwhelmed unless the pool size is decreased. This is because induced mutation detection would require recognition of the mutation in 1 of 32 genomes or 1 of 48, respectively as opposed to 1 of 16 in a heterozygous diploid pool.

Other issues to consider carefully when designing a TILLING or EcoTILLING experiment pertain to the target gene being examined. In general, maximizing the size of the target amplicon (~1 Kb – 1.5 Kb) gives an increased probability of detecting cleaved fragments, especially when working with highly conserved exons or a species that contains a narrow genetic base. Several research papers have previously reported the difficulty of tracking SNPs that are on the ends ~100-200 bp of the target sequence [27, 35, 55, 66, 67, 73]. SNPs detected in our experience in TILLING or EcoTILLING experiments also proved to be difficult to detect when on the ends of the target. One way to avoid this problem is to design primers to target an area slightly larger than the exon so that the ends of the fragment include ~100bp of the intron. Alternatively, one could design multiple primer sets for a particular exon of interest such that these amplicons overlap by at least 100 bases [55]. Additionally, another potential difficulty in EcoTILLING is that as the number of SNPs detected per fragment increases, the scoring and tracking of cleaved fragments becomes more difficult. Single SNPs detected in a heteroduplex produce a higher concentrated product compared to multiple mismatch sites [55, 66]. One needs to be careful scoring images when a large number of SNPs are present in a gene fragment. 

Another point for consideration when designing an EcoTILLING or TILLING experiment is the choice of the nuclease used to digest the mismatches in the heteroduplexes. CEL I, which is derived from celery, is an endonuclease that recognizes and cleaves mismatches in a heteroduplex and also contains 5’ to 3’ exonucleolytic activity [12, 75]. Therefore, CEL I not only digest mismatches in a heteroduplex but also will digest the full length PCR product starting with the 5’ fluorescent label. Therefore, care needs to be taken not to over digest DNA samples, and thus, lose the fluorescent signal of the PCR products. In our TILLING and EcoTILLING work, the mismatch enzyme CEL I was purchased from Transgenomic, Inc. (Omaha, NE) instead of purifying it in the laboratory. This enzyme has an optimal DNA concentration range suggested for efficient digestion. Therefore, in our studies amplicons were quantified and an aliquot of the reaction fitting the target range was used to ensure an efficient digestion. If one decides to purify CEL I or other endonucleases, which can reduce some of the cost for the experiment, then optimum digestion conditions and appropriate enzyme concentrations for enzymatic cleavage should be determined empirically [41, 78]. Other nucleases such as mung bean nuclease, S1, and ENDO1 are either available for purchase or can be purified in the laboratory and have previously been tested for efficient mismatch digestion ability [45, 78].

The last challenge to consider is assigning a particular phenotype to a genotype and inferring the putative function of a gene. Chemical mutagensis is known to introduce background mutations, which can at times make phenotype analysis difficult [30]. This may require several generations of outcrossing or backcrossing [12, 30] to ascertain. Generally, outcrossing M_3_ plant lines may be unnecessary to attribute a phenotype to a mutant genotype. One can evaluate the M_3_ lines generated from a heterozygous selfed M_2_ plant for typical Mendelian segregation ratios, which should allow a correlation between genotype and phenotype. Background mutations, if unlinked to the gene of interest, can be distinguished because they will segregate in the M_3_ generation [12]. Another strategy is to cross two independent TILLED lines to produce heteroallelic individuals so that background mutations will independently assort [12, 30]. Of course, if there is any epistasis or pleiotropic effects from the background mutations or other wild type alleles then assigning a function will be more problematic. Another concern for polyploid plants is that double or triple mutants may be necessary to assign a phenotype to genotype [47] because redundant copies of loci in the genome can mask expression [50].

## GERMPLASM COLLECTIONS AND MAINTAINING GENETIC STOCKS

Due to the current global human population density that is estimated at 6.65 billion and predicted to grow to 9 billion by 2050, maintaining plant and animal germplasm collections both nationally and internationally is essential for supplying food for future generations. In current agricultural practices, crops and livestock tend to be genetically uniform which makes them extremely vulnerable to new diseases or environmental stresses. Preservation of genetic resources allows researchers to breed new combinations of genes that can resist disease, survive in adverse conditions, or provide new desirable traits such as enhancing the nutritional value of food for human consumption. Historically, many heirloom plants and undomesticated animals have been forever lost due to population encroachment, war, natural disaster, climate change, or lack of financing to properly maintain and preserve materials, especially in developing countries. Several germplasm repositories, both federally funded, such as Svalbard Global Seed Vault and grass root organizations across the globe are diligently pursuing novel genetic sources to safeguard these valuable resources and preserve them for future generations. 

Germplasm repositories can be beneficial resources for research scientists for TILLING and EcoTILLING experiments by providing a source to help fill genetic gaps and / or acquire material needed for a study. On the other hand, TILLING could be applied in association / collaboration with germplasm repositories to develop mutant lines that have beneficial traits for breeders, such as improved drought tolerance in plants. Currently, collaborative effort between two U.S. germplasm units (USDA-ARS PGRCU and USDA-ARS Plant Stress and Germplasm Development Unit) includes analyzing sorghum mutant populations to study functional genomics. Mutagensis can potentially lead to the development of improved varieties more rapidly than conventional breeding efforts.

Genetic stocks created by chemical mutagensis (or other approaches) may be beneficial not only for linking a genotype to a phenotype, but also to develop germplasm resources for breeding or obtaining new agronomically important traits. Furthermore, if the genetic stocks are created by chemical mutagensis rather than transformation methods, then extensive regulatory issues may not apply in getting products to the marketplace. Due to the increase in genomic data and availability of high throughput reverse genetic methods, many species of plants and animals have numerous genetic stocks available for breeding purposes or genetic research. Given current trends of the increasingly fast pace collection of sequence data and the desire to understand the function of all the genes, the generation of new genetic stocks will continue to grow in the years to come. Many of these mutants are maintained by species specific centers that house and catalogue the genetic information and distribute the material to requestors / researchers. Preserving this material for breeding and future research is crucial because this material could be utilized to positively impact and improve cultivated germplasm. 

A potential concern, however, is the efficient utilization and overall management of genetic stocks and wild germplasm in centers that house both types of material. In other words, is it more appropriate to maintain genetic stocks and natural wild germplasm separately or together? An advantage to housing genetic stocks within germplasm repositories is that a single source will be available to supply material and necessary information on mutant and wild germplasm. In contrast, a disadvantage to keeping this material separate is loss of valuable information of the collected material when genetic stock research scientists retire or leave for other opportunities, especially in situations in which genetic stocks and associated information is not maintained in a public access database or a species specific center. On the other hand, if genetic stocks are maintained with wild germplasm collections then some collections may grow to a point where they are too large for curators to handle and manage effectively. If this occurs, at some point in time, it may become necessary to evaluate the genetic diversity of these species-specific genetic stocks and maintain only those that appear to be truly novel.

## PERSPECTIVE

TILLING and EcoTILLING have been proven to be highly effective reverse genetic tools for functional genomic studies in plants and animals. Since the inception of these techniques, many researchers have gained indispensable insight on gene function and have identified natural and induced variants. These methods are now well established for many model plant and animal systems regardless of their mating system, genome size, or ploidy level. TILLING is one of the few reverse genetic applications that has not been proven to be applicable in a species specific manner unlike other approaches (i.e.-RNAi or homologous recombination), which potentially makes this application available for all species. The main limitation for TILLING is that the species is capable of being mutagenized. Therefore, for ethical reasons TILLING should not be employed for analyzing functional genomics in humans.

Several laboratory sites have established TILLING and / or EcoTILLING centers for community users as a public service. Currently, TILLING service centers are available (or will be available in the near feature) for *Arabidopsis thaliana*, maize (*Zea mays*), rice (*Oryza sativa*), *Medicago truncatula*, *Lotus japonicus*, *Brassica napus*, tomato (*Solanum lycopersicum*), soybean (*Glycine max*), *C. elegans*, and *Drosophila*. Many of these aforementioned species already have complete genomic information publicly available so the focus for these species has shifted from genomics to empirical determination of gene function. As time goes on, more genomic information will become readily available for other plant and animal species, and thus, reverse genetics approaches will be necessary to assign putative gene function. This aspiration of geneticists to unravel and elucidate the function of coded DNA may eventually lead to the development of public TILLING / EcoTILLING services in numerous plant and animal species, which will facilitate streamlining the process of functional genomics for all researchers.

## Figures and Tables

**Table 1.** Overview of the Published Mutation Frequencies, Mutagen Dose, Genome Size, and Ploidy Level Reported from TILLING Studies in Various Organisms Including Plants and Animals

Organism	Genus Species	Mutagen Applied	Mutagen Dose	Estimated Genome Size	Ploidy	Reported Mutation Rate	Citation
Arabidopsis	*Arabidopsis thaliana*	EMS	20-40 mM	125 Mb	2X	1/300 kb	[27]
Barley	*Hordeum vulgare*	EMS	20-30 mM	5,300 Mb	2X	1/Mb	[48]
Maize	*Zea mays*	EMS	0.0625%	2,500 Mb	2X	0.93/kb B73	[47]
Maize	*Zea mays*	EMS	0.0625%	2,500 Mb	2X	2.10/kb W22	[47]
Maize	*Zea mays*	EMS	1%	2,500 Mb	2X	2/Mb	[46]
Pea	*Pisum sativum*	EMS	4 mM	4,300 Mb	2X	1/669 kb	[45]
Rice	*Oryza sativa*	EMS	1.5%	430 Mb	2X	1/294 kb	[35]
Rice	*Oryza sativa*	Az-MNU	1 mM Az-15 mM MNU	430 Mb	2X	1/265 kb	[35]
Rice	*Oryza sativa*	EMS	0.8-1%	430 Mb	2X	0.5/Mb	[54]
Rice	*Oryza sativa*	EMS	1.6%	430 Mb	2X	1/Mb	[54]
Soybean	*Glycine max*	NMU	2.5 mM	1,115 Mb	2X	1/140 kb	[16]
Soybean	*Glycine max*	EMS	50 mM	1,115 Mb	2X	1/250 kb	[16]
Soybean	*Glycine max*	EMS	40 mM	1,115 Mb	2X	1/550 kb	[16]
Wheat	*Triticum turgidum* subsp. *durum*	EMS	0.75-1%	12,000 Mb	4X	1/40 kb	[51]
Wheat	*Triticum aestivum*	EMS	0.75-1.2%	17,000 Mb	6X	1/24 kb	[51]
Fruit fly	*Drosophila melanogaster*	EMS	50 mM	180 Mb	2X	1/156 kb	[60]
Fruit fly	*Drosophila melanogaster*	EMS	125 mM	180 Mb	2X	1/90.5 kb	[60]
Fruit fly	*Drosophila melanogaster*	EMS	50 mM	180 Mb	2X	1/209 kb	[61]
Nematode	*C. elegans*	EMS	0.025 M	100 Mb	2X	1/293 kb	[33]
Zebrafish	*Danio rerio*	ENU	3.0 mM	1,700 Mb	2X	1/235 kb	[32]

**Table 2.** A List of the Number of Target Genes and Different Classes of Mutations Detected by TILLING from Previously Published Plant and Animal Studies

Organism	No. of Genes	No. of Silent Mutations	No. of Missense Mutations	No. of Nonsense Mutations	No. of Splice Site Mutations	Non Coding	Total	Citation
Arabidopsis	192	851	946	93	--	--	1,890	[27]
Barley	2	4	6	0	0	--	10	[48]
Lotus	1	--	6	--	1	--	15	[43]
Lotus	4	8	28	2	0	19	57	[44]
Maize	11	7	10	0	0	--	17	[46]
Pea	5	19	39	2	0	--	60	[45]
Rice	10	19	29	1	--	8	57	[35]
Soybean	7	42	62	3	0	9	116	[16]
Wheat	3	--	84	3	5	--	246	[51]
Fruit fly	3	10	33	1	0	--	44	[60]
Fruit fly	1	6	6	0	0	4	16	[61]
Nematode	10	27	42	2	0	--	71	[33]
Zebrafish	16	63	119	14	7	52	255	[32]
